# Specific humoral response in cancer patients treated with a VEGF-specific active immunotherapy procedure within a compassionate use program

**DOI:** 10.1186/s12865-020-0338-4

**Published:** 2020-03-14

**Authors:** Javier Sánchez Ramírez, Yanelys Morera Díaz, Mónica Bequet-Romero, Francisco Hernández-Bernal, Yenima Martín Bauta, Katty-Hind Selman-Housein Bernal, Ana Victoria de la Torre Santos, Mariela Pérez de la Iglesia, Lian Trimiño Lorenzo, Team of Investigators of Compassionate use Program, Jorge Víctor Gavilondo Cowley, Miladys Limonta Fernández, Sheila Padrón Morales, Giselle Leal Alpízar, Duneidis Godínez Díaz, José Luis Rodríguez Reinoso, Grettel Melo Suárez, Verena Lucila Muzio González, Luis J. López Carrazana, Ihosvany Enrique Carreño Rolando, Yamirka Sánchez Ascuy, Marta Ayala Avila

**Affiliations:** 1 Department of Pharmaceuticals, Center of Genetic Engineering and Biotechnology (CIGB), Playa, 10600 Havana, Cuba; 2 Department of Clinical Research, CIGB, Playa, 10600 Havana, Cuba; 3 Center of Medical and Surgical Research (CIMEQ), Playa, 12100 Havana, Cuba; 4 “Celestino Hernández” Hospital, 50100 Santa Clara, Villa Clara Cuba; 5 Development Unit, CIGB, Playa, 10600 Havana, Cuba

**Keywords:** CIGB-247, VEGF cancer vaccine, Specific active immunotherapy, Humoral response, Compassionate use program, Cancer patients

## Abstract

**Background:**

CIGB-247 is a cancer therapeutic vaccine that uses as antigen a variant of human vascular endothelial growth factor (VEGF) mixed with the bacterially-derived adjuvant VSSP. CIGB-247 has been already evaluated in two phase I clinical trials (CENTAURO and CENTAURO-2), showing to be safe and immunogenic in advanced cancer patients selected under well-defined and controlled clinical conditions. Surviving patients were submitted to monthly re-immunizations and some of them showed objective clinical benefits. Based on these results, a compassionate use program (CUP) with CIGB-247 was initiated for patients that did not meet the strict entry criteria applied for the CENTAURO and CENTAURO-2 clinical trials, but could potentially benefit from the application of this cancer therapeutic vaccine.

**Results:**

Polyclonal IgM, IgA and IgG antibodies specific for VEGF were detected by ELISA in serum samples from patients vaccinated with 400 μg of antigen combined with 200 μg of VSSP. Polyclonal antibody response showed no cross reactivity for other VEGF family member molecules like VEGF-C and VEGF-D. Serum from immunized individuals was able to block the binding of VEGF to its receptors VEGFR2 and VEGFR1. IgG fraction purified from immune sera shared the aforementioned characteristics and also inhibited the interaction between VEGF and the therapeutic recombinant antibody bevacizumab, an anti-angiogenic drug approved for the treatment of different tumors. No serious adverse events attributable to CIGB-247 have been documented yet in participants of the CIGB-247 CUP.

The present paper is a first report of our findings concerning the humoral response and safety characteristics in treated CIGB-247 CUP cancer patients. The study has provided the unique opportunity of not only testing CIGB-247 in a broader clinical spectrum sample of Cuban cancer patients, but also within the context of the day-to-day clinical practice and treatment settings for these diseases in Cuban medical institutions.

**Conclusions:**

The CIGB-247 CUP has demonstrated that immunization and follow-up of a variety of cancer patients, under day-to-day clinical practice conditions in several Cuban medical institutions, replicate our previous findings in clinical trials: CIGB-247 is safe and immunogenic.

## Background

Vascular endothelial growth factor A (hereafter denominated VEGF) has been broadly studied due to its relevant role in physiological and pathological angiogenesis [1]. During malignant tumor development and progression, cancer cells produce VEGF, among other pro-angiogenic factors, to compensate hypoxia and promote proliferation. Pathological angiogenesis is also important for cancer dissemination and metastases, and for the resistance of tumor cells to the natural immune response. The biological activity of VEGF is mediated by its binding to VEGFR receptor 2 (VEGFR2) or VEGF receptor 1 (VEGFR1) [2]. Both interactions have been implicated in tumor-induced angiogenesis and immunosuppression [3, 4].

All these properties made VEGF an attractive target for cancer immunotherapy. Passive and active immunotherapies targeting this molecule have been developed, and are currently in different stages of preclinical or clinical development. So far, the most successful passive immunotherapy directed to VEGF is bevacizumab, a recombinant monoclonal antibody that neutralizes the binding of VEGF to its receptors [5]. In combination with chemotherapy, bevacizumab has been approved in many countries for the treatment of different tumors [6–12].

VEGF-targeted active immunotherapies are based on different approaches, from DNA to peptide or protein-based active immunotherapeutic procedures [13, 14]. Only the latter has been clinically evaluated. Our group has developed an active immunotherapy procedure (CIGB-247) that uses as antigen a recombinant mutated version of human VEGF genetically coupled to the first 47 aminoacids of the p64K protein. Discrete aminoacid mutations, in the receptor-binding domain sequence of VEGF, were made with the aim of blocking the binding between the antigen and VEGFR2, and hence avoiding any CIGB-247 potential proangiogenic activity [15]. The antigen is formulated with a bacterially-derived adjuvant VSSP [16]. CIGB-247 has shown anti-tumor and anti-metastatic effects in mice, stimulating the development of VEGF-blocking antibodies and specific T cell responses [15, 17]. CIGB-247 has been already evaluated in two phase I clinical trials known as CENTAURO and CENTAURO-2, where safety and immunogenicity were studied in patients with advanced solid tumors [18, 19].

The results obtained from these two trials demonstrated the excellent safety profile of CIGB-247, and also indicated that it is possible to induce a polyclonal antibody response against human VEGF, characterized by the presence of specific IgG, IgM and IgA antibodies. This specific polyclonal antibody response was able to inhibit the interaction between VEGF and its receptors and reduce VEGF bioavailability within platelets [18, 19].

Wentink et al. have also developed a therapeutic vaccine that uses as antigen a truncated form of human VEGF (aminoacids 26–104). This antigen sequence represents the complete bevacizumab binding site. Using RFASE as adjuvant, the vaccine (hVEGF_26–104_/RFASE) induces an immune response with VEGF neutralizing activity and anti-tumor effect [20]. In rats and monkeys immunized with this vaccine candidate, the VEGF-specific polyclonal antibody response has also demonstrated its capacity to impair the binding of bevacizumab to VEGF, suggesting the presence of antibodies that target the same VEGF epitope as bevacizumab [20, 21]. This vaccine is being investigated in a phase I open-label clinical trial (NCT02237638), and preliminary results of the first included patients indicated that hVEGF_26–104_/RFASE has a good safety profile. However, no VEGF-specific antibody responses were found in any of the patients evaluated [22].

As mentioned before, patients enrolled in the CENTAURO clinical trial were in frank progression and they had previously received all available therapies and were no longer responding [18]. After the end of the trial period (week 16), CENTAURO surviving patients were voluntarily enrolled to receive off-trial monthly re-immunizations with CIGB-247 and participate in regular immunological studies. In these individuals we have been able to show an excellent safety profile and sustained specific immune responses [23, 24]. As immunizations have increased over the years, anti-VEGF IgG polyclonal antibody response shifts gradually in some patients from IgG1 to IgG4 [19, 24]. Objective clinical benefits have been documented in a number of surviving CENTAURO patients [23, 24].

All the aforementioned led us to propose to the Cuban Regulatory Authority (CECMED), the initiation of a compassionate use program (CUP) for CIGB-247 in cancer patients that did not meet the strict entry criteria applied for the CENTAURO and CENTAURO-2 clinical trials, but could potentially benefit from the application of CIGB-247. It is well known that a large proportion of cancer patients with life-threatening disease are excluded from clinical trials, despite the fact that these subjects are better reflections of the real-world population of cancer patients [25].

Treating a broader spectrum of cancer patients with CIGB-247 in non-trial conditions would also allow us to continue evaluating the nature, magnitude, persistency of the specific humoral response as well as the safety profile of CIGB-247. To achieve this, cancer patients were vaccinated with 400 μg of antigen combined with 200 μg of VSSP, the highest antigen dose that at that point of the initiation of this program had been found to be safe. Vaccination was administered until death, intolerance, marked disease progression or patient’s withdrawal of consent.

This paper mainly describes and discusses the results of the study of the immune humoral response to CIGB-247 in a sample of the CUP patients treated so far. This work also presents a brief analysis of some safety and product administration aspects that were not possible to assess under the strict patient enrollment criteria applied for the CENTAURO and CENTAURO-2 clinical trials.

## Results

### Characteristics of the patients evaluated during the humoral response study

Table 1 shows the basic characteristics of the CUP patients which had pre and post-vaccination serum samples that allowed the evaluation of the humoral response elicited by CIGB-247.

**Table 1:** Characteristics of the CUP patients for which the humoral response was evaluated

Characteristic	n	Percentage
Age		
≥ 50	30	73%
< 50	11	27%
Sex		
Female	25	61%
Male	16	39%
Primary tumor site ^a^		
Ovary	9	22%
Breast	4	10%
Colon	3	7%
Soft tissues	3	7%
Brain	3	7%
Lymphatic system	3	7%
Lung	2	5%
Kidney	2	5%
Uterus	2	5%
Rectum	2	5%
Others	8	20%
Metastases ^b^		
With metastatic disease	27	66%
Without metastases	14	34%
Metastatic sites ^c^		
Lung	10	26%
Lymph nodes	7	18%
Peritoneum	5	13%
Liver	5	13%
Bone	4	10%
Brain	3	8%
Ovary	2	5%
Others	3	8%

Patients diagnosed with solid tumors or hematologic malignancies were included in the CUP after completing all steps of the recruiting process. ^a^at the time of initial diagnosis; ^b^ metastases at the time of enrollment; ^c^ metastatic sites found in 27 patients with metastatic disease

Of the 41 patients, 25 were female and 16 male (Table 1). Subjects had a variety of malignancies at original diagnosis, being the most common ovary cancer (*n* = 9 for a 22%). At the moment of inclusion in the CUP, 27 patients (66%) had metastatic disease, in some cases localized at multiple sites.

### Antibody classes responses specific for VEGF and VEGF blocking activities of patients’ sera after the induction phase

**Fig. 1:**
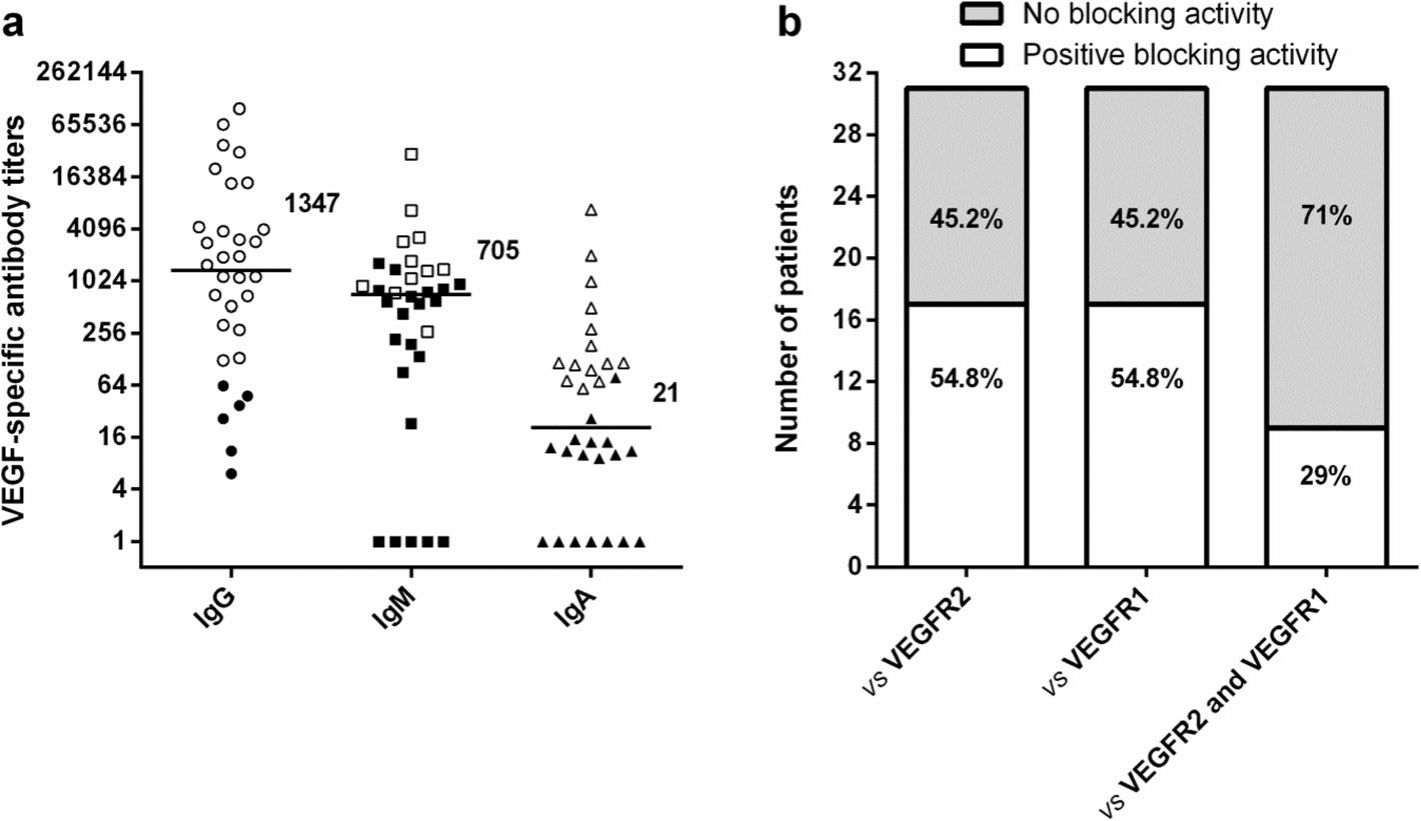
Classes of immunoglobulins specific to VEGF and blocking activity in vaccinated patients during the induction phase. **a** IgG, IgM and IgA specific antibody titers expressed as reciprocal value. Pre-vaccination antibody titer (week 0) was subtracted from the post-vaccination antibody titer (VEGF-specific antibody titer). Post-vaccination serum samples were taken 1 week after the eighth or ninth immunization. Horizontal bars represent the median of specific antibody titer, which are shown for each class of immunoglobulin with its corresponding value. Empty or filled symbols represent patients with positive or negative serum sample, respectively. To declare a given sample taken during vaccination to be positive for antibody, eqs. 1 and 2 were used (**b**) Blocking activity on the VEGF/VEGFR2 or VEGF/VEGFR1 bindings. Patients that has shown at least one serum sample with neutralizing anti-VEGF antibodies were considered with a positive blocking activity. To declare a given sample taken during vaccination to be positive, eq. 4 was used

In order to evaluate the vaccine-induced polyclonal humoral immune response, IgG, IgM, IgA and IgE antibodies specific to VEGF were assessed by enzyme-linked immunosorbent assay (ELISA). Thirty-two patients had at least one serum sample belonging to the induction phase, taken 1 week after the eighth or ninth immunization. Figure 1a displays specific antibody titers against VEGF for the IgG, IgM and IgA classes, respectively, in these subjects. Each patient is represented as an empty symbol (serum sample positive for antibody) or filled symbol (serum sample negative for antibody). It can be seen that of the 32 evaluated patients, 26 individuals (81.3%) had positive samples for VEGF-specific IgG antibodies, 14 (43.8%) for IgA, and 11 (34.8%) for IgM. No patient had detectable levels of VEGF-specific IgE antibodies during the induction phase (Additional file 1). Specific IgG antibodies were found in the highest number of patients, and this type of immunoglobulins showed the highest antibody titers (Fig. 1a).

After the induction phase, there were 9 patients with triple-positive samples (IgG^+^/IgM^+^/IgA^+^), 5 patients with IgG^+^/IgA^+^ double-positive samples and 2 patients with IgG^+^/IgM^+^ double-positive samples. The combination IgM^+^/IgA^+^ double-positive samples was not observed, and all cases with single-positive samples were IgG (10 patients). Single-positive samples for IgM or IgA were not detected, and 6 individuals were triple-negative (IgG^−/^IgM^−^/IgA^−^) (Additional file 1).

In order to investigate the ability of vaccine-elicited antibodies to block the binding of VEGF with VEGFR2 and VEGFR1, a competitive ELISA was performed. Figure 1b shows the number of patients with positive blocking activity on the binding of VEGF with VEGFR2 or VEGFR1. Individuals showing at least one serum sample with neutralizing anti-VEGF antibodies were considered positive for blocking activity. Of the 31 available patients and during the induction phase, 17 patients (54.8%) had a positive blocking activity on the VEGF/VEGFR2 or VEGF/VEGFR1 interactions. Among these patients, 9 subjects (29%) developed a polyclonal antibody response with the ability to simultaneously block the VEGF/VEGFR2 and VEGF/VEGFR1 bindings (dual blocking activity).

These results demonstrate that vaccination with 400 μg of antigen in combination with 200 μg of VSSP induces a polyclonal antibody response comprised by VEGF-specific IgG antibodies as predominant immunoglobulin, but also IgM and IgA antibodies can be detected. Elicited antibodies also block the interaction between VEGF and its receptors VEGFR2 and VEGFR1.

### Specific anti-VEGF IgG seroconversion and VEGF blocking activities of patients’ sera during the re-immunization phase

In order to investigate whether repetitive immunizations help to maintain a systemic humoral response directed to human VEGF, IgG seroconversion and blocking activity were analyzed in serum samples at different time points during the re-immunization phase. The re-immunization phase comprised from week 16 to week 135 (≈ 2.8 years) and re-immunizations were administered every 4 weeks.

**Fig. 2:**
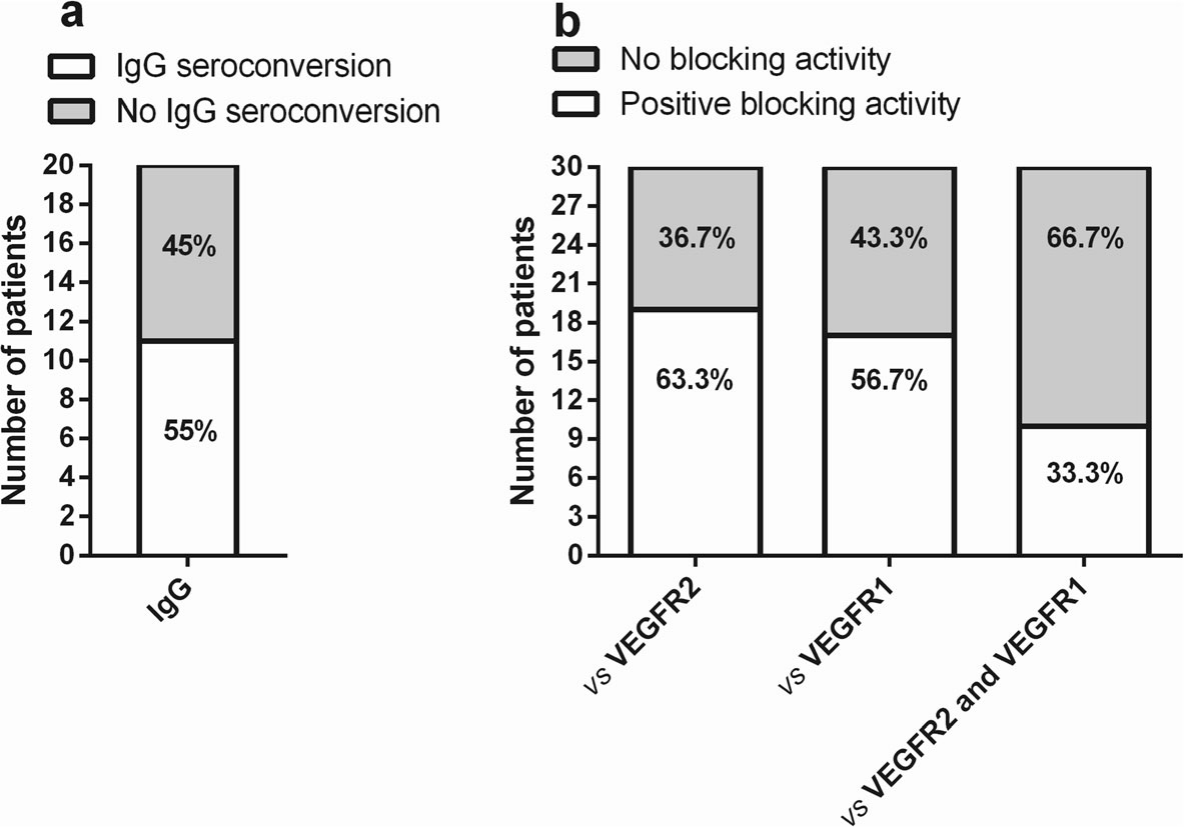
Anti-VEGF IgG seroconversion study and serum blocking activity in vaccinated patients during the re-immunization phase. **a** Seroconverted patients (individual that has shown two or more samples positive for VEGF-specific IgG antibodies). **b** Blocking activities on VEGF/VEGFR2 or VEGF/VEGFR1 bindings. Patients that has shown at least one serum sample with neutralizing anti-VEGF antibodies were considered with a positive blocking activity

Of the 30 patients available during the re-immunization phase, 20 individuals had at least two serum samples after week 16. Figure 2a shows the number of patients with specific anti-VEGF IgG seroconversion. Among these subjects, 11 patients (55%) were classified as seroconverted for VEGF-specific IgG antibodies, while the remaining patients (45%) did not achieve this status (Fig. 2a).

Also, serum samples from 30 patients were available for analysis of the blocking activity against the binding between VEGF and its receptors. Figure 2b shows the number of patients with positive blocking activity on the binding of VEGF with VEGFR2 or VEGFR1. Among these patients, 19 (63.3%) or 17 (56.7%) individuals showed a positive blocking activity for VEGF/VEGFR2 or VEGF/VEGFR1 interactions, respectively. Among these patients, 10 individuals (33.3%) had dual blocking activity (Fig. 2b).

### IgG subclasses during the study

**Fig. 3:**
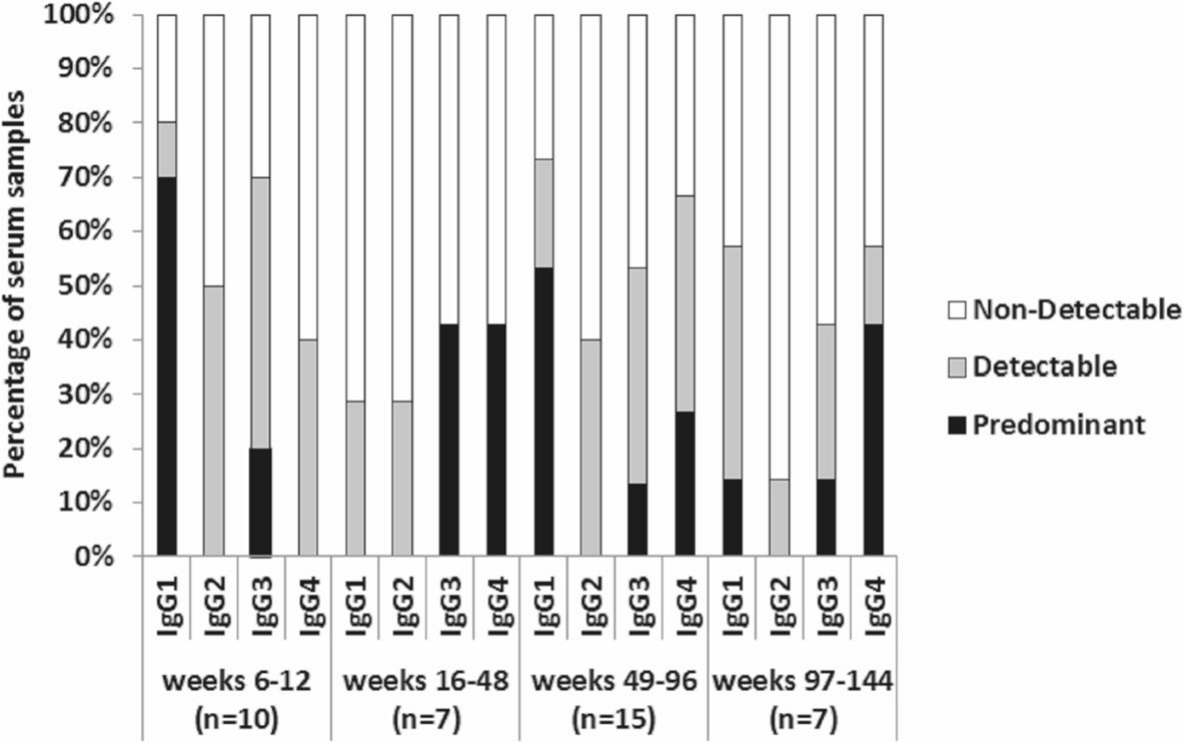
VEGF-specific IgG subclasses between weeks 6–12, 16–48, 49–96 and 97–144. The study was made for the available serum samples classified as positive for VEGF-specific IgG antibodies. “n” represents the number of the available serum samples. Terms “non-detectable”, “detectable” and “predominant” are detailed in Methods

Within the polyclonal antibody response directed to VEGF, IgG is the principal immunoglobulin found. In order to study the contribution of each one of the four VEGF-specific IgG subclasses, an indirect ELISA was performed using human VEGF as coating antigen. Figure 3 shows IgG subclasses analysis in four different vaccination periods: weeks 6–12, weeks 16–48 (up to 1 year), weeks 49–96 (up to 2 years) and weeks 97–144 (up to 3 years). In each of these periods, available serum samples classified as positive for VEGF-specific IgG antibodies were chosen for these measurements. IgG1, IgG2, IgG3 and IgG4 subclasses specific to VEGF were found in all periods. IgG1 was the predominant subclass during the induction phase, accounting for 70% of the available serum samples, and IgG3 was the second most important immunoglobulin with 20% (Fig. 3). Between weeks 16–48, IgG3 and IgG4 subclasses were the predominant subclasses with 43% of the available serum samples. However, IgG1 was become the predominant subclasses between weeks 49–96, accounting for 53% of the available serum samples, and IgG4 was the second most important immunoglobulin with 27%. After 2 years of repetitive immunizations, between weeks 97–144, IgG4 was the predominant subclass with 43% of the available serum samples (Fig. 3).

### Properties of the IgG fraction purified from the serum of vaccinated patients

In order to assess whether the immunological properties detected in immune serum (specificity for VEGF and blocking activity) were shared by its IgG fraction, post-vaccination serum samples classified as positive for VEGF-specific IgG antibodies were pooled, and IgG immunoglobulins were purified (IgG comp).

**Fig. 4:**
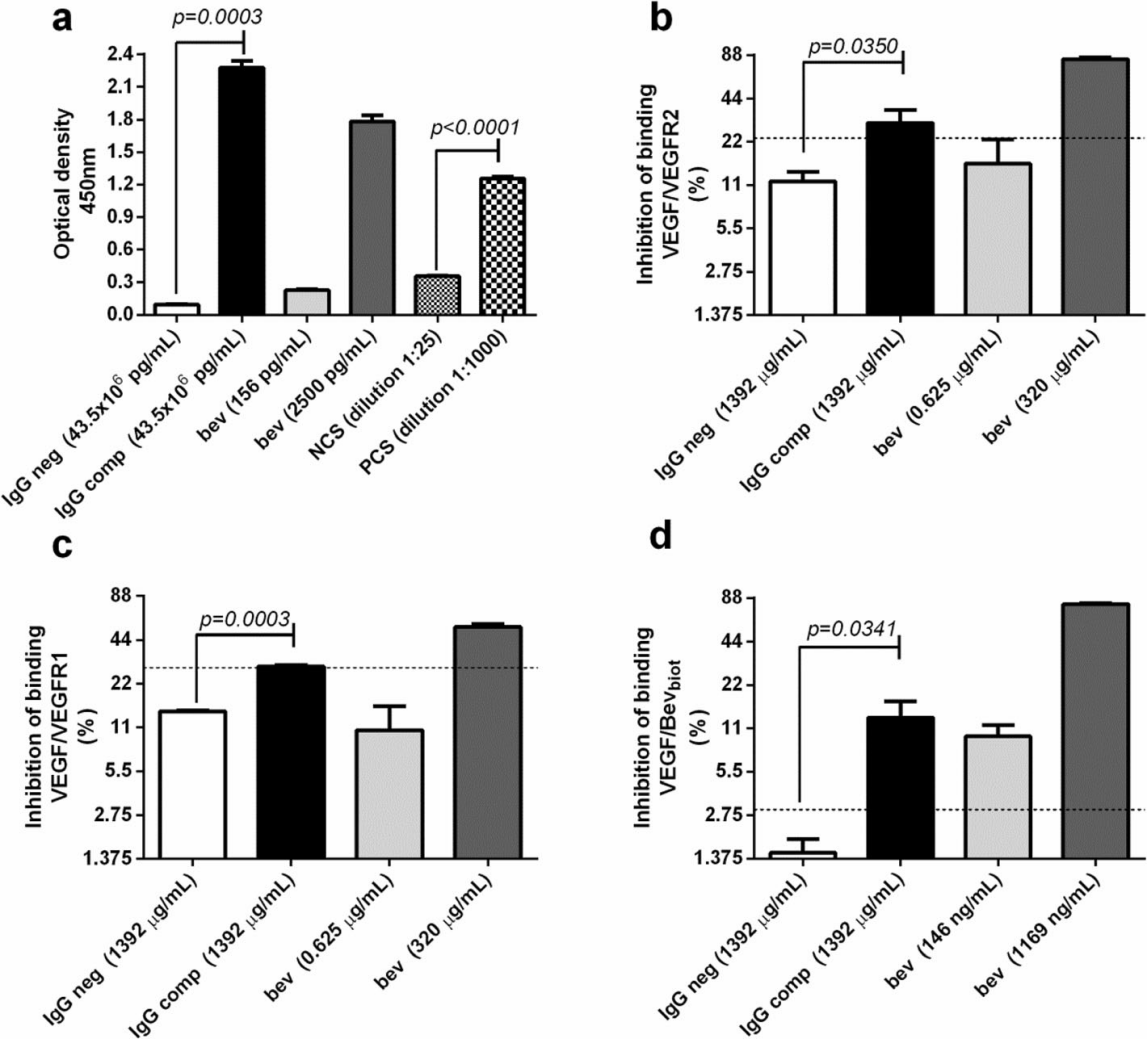
Properties of the IgG fraction purified from de serum of vaccinated patients (IgG comp). **a** Binding of IgG to VEGF-coated wells. Test samples were added to VEGF-coated wells and IgG bound to wells was detected with HRP-conjugated goat anti-human IgG antibody. This assay used as positive controls a human serum positive for VEGF-specific IgG antibodies (PCS) and bevacizumab (bev). Assay negative controls were a human serum negative for VEGF-specific IgG antibodies (NCS) and a purified human IgG isolated from pooled normal human serum (IgG neg) (**b**) Inhibition of the VEGF/VEGFR2 interaction. Test samples and VEGFR2-Fcγ were added to VEGF-coated wells, and VEGF/VEGFR2-Fcγ binding was determined with biotin-conjugated anti-VEGFR2 antibody followed by streptavidin-peroxidase conjugate (**c**) Inhibition of the VEGF/VEGFR1 interaction. Test samples and VEGFR1-Fcγ were added to VEGF-coated wells, and VEGF/VEGFR1-Fcγ binding was determined with biotin-conjugated anti-VEGFR1 antibody followed by streptavidin-peroxidase conjugate (**d**) Inhibition of the VEGF/bevacizumab interaction. Test samples and biotinylated bevacizumab (bev _biot_) were added to VEGF-coated wells, and VEGF/Bev _biot_ binding was determined with streptavidin-peroxidase conjugate. Discontinued lines represent cut-off values that define the positivity for VEGF/VEGFR2, VEGF/VEGFR1 or VEGF/Bev blockade. The ability of test samples to block these three interactions was expressed as percentage of reduction of the maximum binding. Bev was used as inhibition positive control. IgG comp and IgG neg were evaluated in all ELISAs at the same total IgG concentration. All figures show the response obtained for IgG comp that exhibited the best ratio IgG comp/IgG neg. Column bars represent the means of three replicates and error bars represent standard deviations. *p*-values were calculated according to unpaired *t*-test. Statistical significance was considered as *p* < 0.05

Figure 4a shows that immobilized VEGF was recognized by purified IgG (IgG comp) and by the assay positive controls (bevacizumab and positive control serum (PCS)). Low levels of binding were observed for negative IgG (IgG neg) and negative control serum (NCS) (Fig. 4a). VEGF-specific IgG antibodies detected in IgG comp were significantly higher than those found in IgG neg (unpaired *t* test, *p* = 0.0003). Similar results were observed between PCS and NCS (unpaired *t*-test, *p* < 0.0001).

For the interaction between immobilized VEGF and VEGFR2, IgG comp exhibited an inhibition value of 29.88%, while IgG neg, at the same total IgG concentration, showed an inhibition value of 11.70%. IgG comp had a significantly higher blocking activity in relation to the blocking activity seen for IgG neg (unpaired *t*-test, *p* = 0.0350) (Fig. 4b). For the binding of VEGF with VEGFR1, IgG comp exhibited an inhibition value of 28.98%, while IgG neg, at the same total IgG concentration, showed an inhibition value of 14.20%. Blocking activity detected in IgG comp was significantly higher than the activity found in IgG neg (unpaired *t* test, *p* = 0.0003) (Fig. 4c). A positive blocking activity on the interaction between VEGF and its receptors was detected for IgG comp, showing in both cases, inhibition levels greater than 2 (see eq. 4). Both competitive ELISAs used bevacizumab as assay positive control at different concentrations, indicating that the test is able to detect changes on inhibition percentages.

In order to investigate whether the polyclonal response elicited by CIGB-247 includes antibodies able to block the VEGF epitope relevant for bevacizumab binding, a competitive ELISA was developed using this recombinant monoclonal antibody as competitor. IgG neg showed an inhibition value of 1.51%, while IgG comp, at the same total IgG concentration, showed an inhibition value of 13.06%. Blocking activity detected in IgG comp was significantly higher than the activity found in IgG neg (unpaired *t* test, *p* = 0.0341) (Fig. 4d). A positive blocking activity (inhibition levels greater than 2) on the interaction between VEGF and bevacizumab was observed for IgG comp.

All results presented so far indicate that the immunological properties described for serum of vaccinated cancer patients (specificity for VEGF and dual blocking activity) can be reproduced by the purified IgG fraction. As additional element, the polyclonal response induced by CIGB-247 comprised bevacizumab-blocking antibodies.

### Cross-reactivity of the CIGB-247 human polyclonal antibody response with VEGF family members

In order to explore whether the VEGF-specific polyclonal antibody response recognizes VEGF-C or VEGF-D through their homology domains, two ELISA formats were developed.

**Fig. 5:**
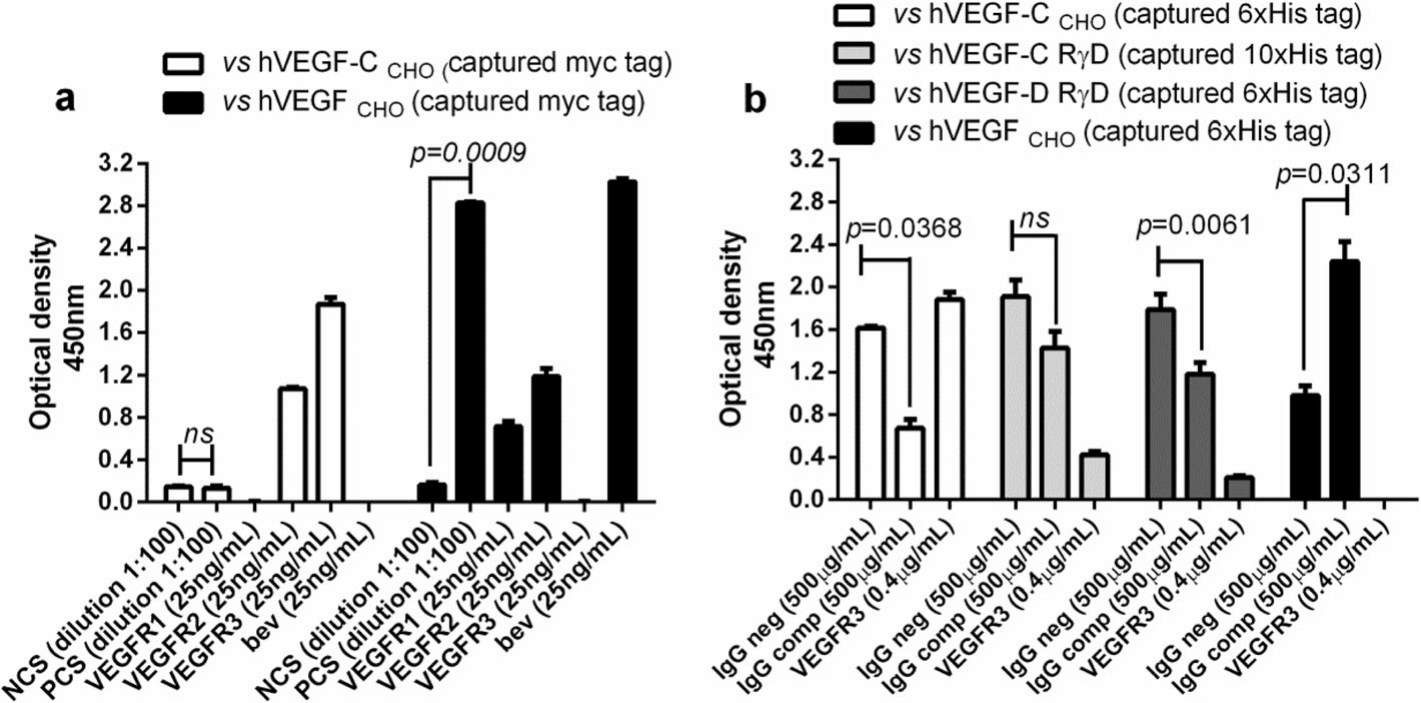
Binding experiments using as ligands human VEGF-C, human VEGF-D and human VEGF. **a** Binding to VEGF-C or VEGF of different types of samples with specificity for VEGF. Myc-tagged proteins, VEGF-C or VEGF obtained from CHO cells (hVEGF-C _CHO_ or hVEGF _CHO_, respectively), were captured using coated wells with a monoclonal antibody specific to myc-tagged proteins. Test samples were added and the binding of IgG or VEGFR1-Fcγ or VEGFR2- Fcγ or VEGFR3-Fcγ was detected with HRP-conjugated goat anti-human IgG antibody. This assay used as test samples a human serum positive for VEGF-specific IgG antibodies (PCS) and a human serum negative for VEGF-specific IgG antibodies (NCS). Control assays were bevacizumab (Bev) and VEGFR1, VEGFR2 and VEGFR3. **b** Histidine-tagged proteins (hVEGF-C _CHO_, hVEGF _CHO_, or commercially available VEGF-C and VEGF-D) were captured using nickel coated multiwell plates. Test samples were added and the binding of IgG or VEGFR1-Fcγ or VEGFR2- Fcγ or VEGFR3-Fcγ was detected with HRP-conjugated goat anti-human IgG antibody. This assay used as test samples a human IgG purified from a pool of serum belonging to patients classified with positive VEGF-specific IgG antibodies (IgG comp) and a human IgG isolated from pooled normal human serum (IgG neg). This assay used as control VEGFR3. Column bars represent the means of three replicates and error bars represent the standard deviations. *p*-values were calculated according to unpaired *t*-test. Statistical significance was considered as *p* < 0.05. ns non-significant

When hVEGF-C _CHO_ is captured with a monoclonal antibody specific to myc-tagged proteins, the optical density observed for PCS and NCS was similar and without statistically significant differences (unpaired *t* test, *p* = 0.4944) (Fig. 5a). However, hVEGF-C _CHO_ was recognized by its cognate receptors: VEGFR2 and VEGFR3. As expected, there was no binding of VEGFR1 and bevacizumab (Fig. 5a). To check this form of ligand presentation in ELISA, hVEGF _CHO_ was evaluated in the same ELISA conditions, and VEGF was recognized by PCS with statistically significant differences as compared to NCS (unpaired *t* test, *p* = 0.0009). Also, VEGF was recognized by bevacizumab and its cognate receptors VEGFR1 and VEGFR2. As expected, there was no binding for VEGFR3 (Fig. 5a).

hVEGF-C _CHO_ was recognized by VEGFR3 when was captured through its histidine tag using nickel coated high sensitivity multiwell plates; binding of IgG comp was lower than that observed for IgG neg with statistically significant differences (unpaired *t* test, *p* = 0.0368) (Fig. 5b). Similar result was obtained for commercially available VEGF-D (unpaired *t* test, *p* = 0.0061). For commercially available VEGF-C, no differences were found between IgG comp and IgG neg (unpaired *t* test, *p* = 0.0660). This form of ligand presentation in ELISA was checked using hVEGF _CHO_ at the same conditions. As expected, VEGF was recognized by IgG comp with statistically significant differences as compared to IgG neg (unpaired *t* test, *p* = 0.0311). There was no binding of VEGFR3 to the captured VEGF (Fig. 5b).

The results indicate that CIGB-247 elicits a polyclonal antibody response highly specific for human VEGF, and this antibody response does not cross-react with human VEGF-C and human VEGF-D.

### Safety profile and immunogenicity of CIGB-247 in patients with previously unexplored clinical scenarios

The CIGB-247 CUP made possible the evaluation of cancer patients under onco-specific treatment, and with additional chronic illnesses, very different from the sample of individuals enrolled for the CENTAURO and CENTAURO-2 clinical trials. Patients vaccinated with CIGB-247 and concomitantly treated with chemotherapy, radiotherapy, biological therapies or immune suppressing drugs as well as patients with hematological malignancies (Hodgkin lymphoma), brain primary tumors, brain metastases, autoimmune diseases (systemic lupus erythematosus) or concurrent chronic diseases (diabetes mellitus, arterial hypertension, cardiopathy, bronchial asthma) do not meet entry criteria applied in the CENTAURO and CENTAURO-2 clinical trials. However, the CIGB-247 CUP included patients with these characteristics. Table 2 depicts this for 19 patients representing new and challenging scenarios for the therapeutic intervention with CIGB-247. In the sample, one patient had a brain primary tumor (UC-CQ111), other patient had brain metastases (UC-CH48) and 6 patients had non-cancerous chronic illnesses (UC-CH47, UC-HA14, UC-CH48, UC-CH08, UC-CH38 and UC-CH25). Many had extensive metastatic disease. Twelve patients were treated with chemotherapy, radiation therapy, passive immunotherapy, cytokines, or other onco-specific procedures under immunization with CIGB-247.

**Table 2:** Patients with different clinical conditions vaccinated with CIGB-247 and treated with different cancer therapies

Patient code	Diagnosis	Metastases	Relevant information	Immunogenicity/Safety profile
UC-CH47	Breast carcinoma	Lung	Cardiopathy patient that received CIGB-247 simultaneous with QT (Capecitabine and Letrozol).	Patient positive for anti-VEGF antibody response. Physician did not report any negative incidence on heart disease.
UC-CH77	Breast carcinoma	Bones	Patient that received CIGB-247 simultaneous with QT (Capecitabine, Zoledronic acid) and Trastuzumab	Patient positive for anti-VEGF antibody response.
UC-CH49	Ovary ADC	Peritoneum	Patient that received CIGB-247 simultaneous with QT (Docetaxel, Carboplatin)	Patient positive for anti-VEGF antibody response.
UC-HA03	Ovary ADC	Contralateral ovary	Patient that received CIGB-247 simultaneous with QT (Paclitaxel, Carboplatin) and G-CSF	Patient positive for anti-VEGF antibody response.
UC-HA07	Ovary ADC	Contralateral ovary	Patient that received CIGB-247 simultaneous with QT (Taxol, Cisplatin)	Patient positive for anti-VEGF antibody response.
UC-CQ108	Bladder ADC	Lung	Patient that received CIGB-247 simultaneous with QT (Gemcitabine, Carboplatin)	Patient positive for anti-VEGF antibody response.
UC-CH10	Hodgkin lymphoma	LN	Patient that received CIGB-247 simultaneous with QT (Procarbazine, Vincristine) and prednisone	Patient positive for anti-VEGF antibody response.
UC-CH11	SCLC	Neck, contralateral pulmonary metastases	Patient that received CIGB-247 simultaneous with QT (Cisplatin, Etoposide (VP-16), taxol, vincristine) and G-CSF.	Patient positive for anti-VEGF antibody response.
UC-CQ105	Rectum ADC	–	Vaccination with CIGB-247 was applied simultaneous with QT (Capecitabine)	Patient positive for anti-VEGF antibody response.
UC-HA01	Peritoneum carcinoma	–	Patient that received CIGB-247 simultaneous with QT (Docetaxel, Paclitaxel) and submitted to a major abdominal surgery (phlegmon).	Patient positive for anti-VEGF antibody response. Physician did not report any negative incidence on wound healing.
UC-CH22	Breast carcinoma	Contralateral breast and LN	After fifth vaccination with CIGB-247, patient was submitted to urgency surgery (right tubo-ovarian abscess) and immunization was not interrupted.	Patient positive for anti-VEGF antibody response ^a^. Physician did not report any negative incidence on wound healing.
UC-HA14	Ovary ADC	–	Insulin-dependent diabetic patient with oral administration of metformin	Patient positive for anti-VEGF antibody response. Physician did not report any negative incidence on diabetes condition.
UC-CH48	Ovary carcinoma	Liver, brain, peritoneum	Patient diagnosed with diabetes mellitus	Patient positive for anti-VEGF antibody response. Physician did not report any negative incidence on diabetes condition.
UC-CH08	Malignant pleural mesothelioma	–	Cardiopathy patient with diabetes mellitus	Patient positive for anti-VEGF antibody response. Physician did not report any negative incidence on chronic diseases.
UC-CH38	Osteosarcoma	Lung	Patient with controlled-chronic bronchial asthma	Patient positive for anti-VEGF antibody response.
UC-CH25	Cervix carcinoma	Liver and bones	Patient diagnosed with systemic lupus erythematosus	Patient positive for anti-VEGF antibody response. Physician did not report any negative incidence on autoimmune disease.
UC-CH46	Thyroid carcinoma	LN and lung	Patient that received CIGB-247 simultaneous with oral levothyroxine	Patient positive for anti-VEGF antibody response.
UC-CQ116	RCC	Lung	Patient that received CIGB-247 simultaneous with recombinant IFN-α2b	Patient positive for anti-VEGF antibody response.
UC-CQ111	GBM	–	Patient that received CIGB-247 simultaneous with RT	Patient positive for anti-VEGF antibody response.

^a^Patient positive for anti-VEGF antibody response is referred to subjects that showed at least one serum sample with antibodies specific to VEGF or with VEGF blocking activity detected during induction or re-immunization phases. *ADC* adenocarcinoma, *QT* chemotherapy, *LN* lymph nodes, *RT* radiotherapy, *G-CSF* granulocyte-colony stimulating factor, *SCLC* small cell lung cancer, *GBM* Glioblastoma multiforme, *RCC* Renal cell carcinoma, *IFN* Interferon

No serious adverse events, probably or definitively related to the vaccine were reported in immunized cancer patients with other chronic diseases including diabetes mellitus, arterial hypertension, cardiopathy, bronchial asthma or systemic lupus erythematosus (Table 2). Patients UC-CH22 and UC-HA01 were submitted to urgency surgery during their vaccination with CIGB-247, and showed a normal recovery in terms of internal and external wound healing. Table 2 also indicates that CIGB-247 is able to induce a detectable VEGF-specific antibody response in patients concomitantly treated with chemotherapeutic agents, cytokines, monoclonal antibodies, immunosuppressant drugs and radiotherapy.

## Discussion

CIGB-247 can be considered the most clinically advanced strategy worldwide targeting VEGF via active immunotherapy. The proposed mechanism of action for this intervention is related to the induction of a long lasting, self-regulated, nontoxic response of both humoral and cellular nature [13]. The immune response elicited by CIGB-247 has been extensively investigated in two phase I clinical trials (CENTAURO and CENTAURO-2), with follow up studies in long-term surviving patients [18, 19, 24], with documented clinical benefits in the latter [23]. The CIGB-247 CUP was initiated mainly as a way of potentially benefiting cancer patients that were/are ineligible for the early phase clinical trials that evaluate this vaccine candidate. This means that cancer patients with different stages of their disease, brain primary tumors, brain metastases, additional chronic uncompensated, autoimmune or immune suppressing diseases as well as patients receiving immune modulator drugs, chemotherapy or biological therapies have been recruited for the CUP. The CIGB-247 CUP is developed in medical Cuban institutions, and headed by physicians and specialists trained in clinical trial development. This effort also opened the opportunity of gathering new information about the specific antibody response and safety profiles of CIGB-247 in a broader clinical spectrum sample of cancer patients, treated within the context of day-to-day clinical practice settings.

Some clinical studies on cancer vaccines have revealed a relationship between the high levels of elicited antibodies and an improved survival; and even have been able to elucidate the specific immunoglobulin class associated with overall survival [26–28]. Although these types of correlations are not applicable to this CUP study of CIGB-247, there is no doubt about the importance of studying in depth the humoral response in early evaluations of cancer vaccines in humans [29]. A special emphasis was done in this paper in the characterization of the vaccine-induced humoral response in terms of quantity, quality and composition.

This report confirms some of the results obtained from the CENTAURO and CENTAURO-2 clinical trials, where CIGB-247 induced a polyclonal antibody response against human VEGF with a dual blocking activity on the interactions with VEGFR2 and VEGFR1. The polyclonal antibody response was characterized by the presence of IgM, IgA and IgG antibodies specific to VEGF, being the latter the predominant immunoglobulin [19]. So far VEGF-specific IgE antibodies have not been detected in any of the studies of CIGB-247, and the presence of this class of immunoglobulin has been reported in very few studies of specific active immunotherapy with other antigens and adjuvants [28, 30]. Although IgE has been pointed out as a mediator of anti-tumor effect [31, 32], the generation of IgE antibodies by active immunization is probably the result of the specific antigen, adjuvants or schedules, or a combination of these.

Additionally, this work shows now that the polyclonal antibody response elicited by CIGB-247 is able to block the binding of bevacizumab to VEGF, adding this property to other previously described for the interaction of VEGF with VEGFR2 and VEGFR1 [19].

This VEGF-neutralizing activity is probably caused in a greater part to a steric hindrance, and in a lesser extent to a competition for the same critical binding determinants on VEGF. This hypothesis is based on the fact that CIGB-247 uses as antigen a protein representative of human VEGF 121 isoform, in which amino acids R82, K84 and H86 have been mutated to E in order to prevent possible undesired pro-angiogenic activities [15]. These three residues have been reported as important for the bindings between VEGF with VEGFR2, VEGFR1 and bevacizumab [33–37]. Because of the aforementioned, the blocking activity induced by CIGB-247 against these three molecules could be probably explained mainly due to steric hindrance, and could very well be also at the basis of the inhibition of the interaction of VEGF with VEGFR2, VEGFR1 and bevacizumab. The polyclonal antibody response elicited after immunization blocks the receptor binding domain on natural VEGF as well as the binding site for bevacizumab. The finding of the presence of bevacizumab-blocking antibodies within the polyclonal response elicited by CIGB-247 indicates that the specific humoral response is directed to relevant domains on VEGF. The induction of bevacizumab-blocking antibodies by active immunization has been also developed by Wentink et al. This approach is based on vaccination against the bevacizumab binding site on VEGF using a truncated protein (human VEGF _26–104_) as antigen and RFASE as adjuvant [21]. This VEGF therapeutic vaccine has shown antiangiogenic and antitumor activity in pre-clinical models [20], but discrete results in terms of immunogenicity have been observed in the first cancer patients treated with the vaccine [22].

Because VEGF binding determinants for VEGFR2 and VEGFR1 overlap only partially, and their binding sites are located at opposite ends of the molecule [38] steric hindrance is a suitable explanation for the VEGF-blocking activity detected in the serum of vaccinated patients. In fact, it has been proposed that the neutralizing effect of bevacizumab for VEGF binding to VEGFR2 and VEGFR1 is also based on steric hindrance [37]. The polyclonal nature of the response to CIGB-247 and the showed evolution with immunization time in different individuals, imply that different classes and subclasses of antibodies, with different affinity, avidity and antigen-recognition sites, are present in the patients. The specific polyclonal antibodies elicited by CIGB-247 probably have a more effective VEGF neutralization than that of the monoclonal antibody. The polyclonal nature of the response to CIGB-247 as compared with monoclonal antibody bevacizumab could yield the same levels of VEGF blocking activity with a lower amount of specific antibodies. The potential benefits of a vaccine strategy over a passive monoclonal antibody immunotherapy, in terms of the efficacy of the binding antigen/antibody, have been also outlined by others. An epidermal growth factor receptor 2 (HER 2)-based vaccine has been compared to the monoclonal antibodies trastuzumab and pertuzumab [39], which have been approved for the treatment of HER 2 overexpressed breast cancers. The immunization with the extracellular domain of HER 2 (HER 2-ECD) elicited polyclonal antibodies with specificity for 14 different epitopes. These polyclonal antibodies inhibited the binding of trastuzumab and pertuzumab [39, 40], which target different subdomains of HER 2-ECD. The properties exhibited by HER 2-induced antibodies included higher growth inhibition and significant receptor internalization, not observed when HER 2 overexpressing tumor cells were treated with trastuzumab [39, 40].

To achieve a clinically relevant effect using a cancer vaccine, it is not only important to generate a robust immune response, but also to sustain this response over time with booster vaccinations. On this regard, patients recruited for the CENTAURO and CENTAURO-2 clinical trials received off-trial monthly re-immunizations until death, intolerance, marked disease progression or patient’s withdrawal of consent [18, 19]. This re-immunization phase was important to sustain the VEGF-specific immune response generated during the induction phase [23, 24]. On this basis, individuals participating in the CIGB-247 CUP were also submitted to re-immunizations. In line with our findings in the previous clinical studies, re-immunizations helped to maintain the seroconversion status and blocking activity in most of the patients. Booster vaccination has been broadly used in cancer vaccines [28, 41, 42], indicating the importance of this strategy for the maintenance of the immune response.

During repetitive vaccinations and the generation of an immune response, there is a programmed order of IgG subclass usage. Collins et al. have been proposed a sequential switch where B cells do a first switch from IgM to IgG3, then to IgG1 and to IgG2 and finally to IgG4, although the switch can also occur directly from IgM to a particular IgG subclass. For this mechanism the affinity maturation increases in the following order: IgG3 < IgG1 < IgG2 < IgG4 [43]. The humoral response against VEGF elicited by CIGB-247 has been characterized by the presence of IgG1 as predominant subclass from the induction phase to 1 year of monthly re-immunizations [19]. After long term immunization and up to 3 years, IgG4 is the predominant immunoglobulin [24]. On the other hand, IgG3 subclass has a trend to disappear after 1 year of vaccinations; however it can be detected in some patients after 3 years of long term immunizations [19, 24]. Most of these results have been confirmed in the present study. It is likely that the putatively high affinity VEGF-specific IgG4 antibodies elicited after chronic vaccinations with CIGB-247 have an active role as effective blockers of the binding between VEGF and its receptors VEGFR2 and VEGFR1. In the case of IgG3, this is considered a relatively transient immunoglobulin, commonly associated with a primary immune response after initial exposure to an antigen [43]. The detection of VEGF-specific IgG3 antibodies during the re-immunization phase could be explained by the generation of new B cell clones, ready to initiate a programed process of sequential switching. The generation of new B cell clones during different time points of booster vaccination has been described by other investigators when two healthy donors were immunized with the tetanus toxoid (TT) vaccine [44]. The TT-specific serum IgG repertoire after booster vaccination comprised new clonotypes not observed before and their frequencies varied between subjects.

Despite the fact that since 2011 a thoroughly characterization of the humoral response elicited by CIGB-247 in cancer patients has been performed [18, 19, 24], no data about a possible cross-reactivity of the VEGF-specific polyclonal response with other VEGF family members had been done. The VEGF family is integrated by several members: VEGF, VEGF-B, VEGF-C, VEGF-D, and others less studied like VEGF-E and VEGF-F [45–47]. VEGF is a principal mediator of angiogenesis through its binding to VEGFR2; meanwhile VEGF-C and VEGF-D can also bind to the same receptor and exert their biological activity. The amino acid sequence similarity between VEGF and VEGF-C or VEGF-D is 47 and 35% respectively. Herein presented results indicate that the antibody response induced by CIGB-247 is highly specific to VEGF and does not cross-react with VEGF-C and VEGF-D [48].. The lack of antibody cross-reactivity with molecular families of the antigen has been also reported in other cancer vaccines. For example, epidermal growth factor (EGF) and transforming growth factor-α (TGF-α) belong to EGF family, and both structurally related soluble proteins (between 30 and 40% amino acid homology) exert their action after interaction with a common cell surface EGF receptor [49]. CIMAvax-EGF is a therapeutic vaccine able to induce in humans a polyclonal antibody response directed to EGF, with the ability to block the interaction between EGF and the EGF receptor. However, the presence of antibodies against TGF-α has not been detected in immunized individuals [50]. In another example, Hosseini-Ghatar and colleagues generated three polyclonal antibodies against HER 2-ECD and none were able to bind to the other members of the human HER family [39].

As mentioned before, the CIGB-247 CUP allowed us to evaluate the preparation in cancer patients, under clinical conditions not yet explored before. In our previous clinical trials [18, 19], cancer patients with brain metastases, Hodgkin lymphoma or with other concomitant chronic diseases were not eligible for enrollment. Simultaneous onco-specific treatment and surgical procedures were also exclusion criteria.

In the sample presented in this paper, it is noteworthy that immunization with CIGB-247 was safe and well tolerated in all these cases. In addition to this experimental evidence, a detectable VEGF-specific antibody response was elicited in patients concomitantly treated with different chemotherapeutic agents. These findings provide the basis to further investigate the combination of CIGB-247 with standard-of-care drugs in patients with different types of cancer. Combinations of conventional chemotherapeutics with specific active immunotherapy are known [51–53]. Chemotherapy is generally administered in regimens allowing and sometimes enhancing the development of the immunotherapy-induced response. We have recently initiated a phase II clinical trial with CIGB-247 in advanced ovarian cancer patients, where individuals receive neoadjuvant chemotherapy followed by interval debulking surgery, and all these procedures in concomitancy with immunization (RPCEC00000246).

Finally, two individuals included in our present study were submitted to major urgent surgeries during immunization with CIGB-247 without reports of wound healing impairment. It is know that bevacizumab treatment increases the risk of bleeding and wound healing complications in cancer patients [54]. These clinical observations with CIGB-247 could be explained by the incomplete abrogation of platelet VEGF levels after immunization with CIGB-247 [55]. After vaccination, remaining active VEGF molecules within platelets are probably sufficient to maintain the recovery response in wound healing, normal adult vasculature or other VEGF-dependent normal physiological processes.

In the CENTAURO clinical trial, platelet VEGF levels were evaluated by ELISA test and antigen-specific interferon-gamma (IFN-γ)-secreting cells were also measured by enzyme-linked immunospot assay (ELISPOT) using stimulated-peripheral blood mononuclear cells (PBMC) [18]. This investigation does not show data about the effect of CIGB-247 on platelet VEGF levels and frequency of cytokine-producing T cells, the latter as indicative of the cellular immune response.

Mandatory and programmed blood sample collections were not required in the CIGB-247 CUP, and these elements were optional and subjected to the physician’s criteria, limiting the number of immunogenicity tests. Also, during the routine clinical evaluation of CUP patients, the blood sample collection, processing, handling and storage (− 20 °C) of serum and plasma were suitable for antibody tests (immunoglobulin classes, IgG subclasses and blocking activity vs VEGFR2 and VEGFR1); however these conditions are not appropriate for VEGF quantification in serum and plasma, needing a storage temperature of − 70 °C [55].

On the other hand, between 50 and 60 mL of blood are required for isolation of PBMC by Ficoll density gradient. This volume of blood is at least five times higher than the volume used for routine clinical monitoring of cancer patients. For that reason, no data about cellular immune response elicited by CIGB-247 are shown here. Despite these limitations, ELISA tests used in this CIGB-247 CUP have been of importance for the immunological evaluations performed in all patients included in the CENTAURO and CENTAURO-2 clinical trials and their off-trial follow-up studies [19, 24].

Objective clinical benefits have been previously documented in a number of surviving CENTAURO patients [23, 24]. The information regarding clinical benefits observed in some patients only vaccinated with CIGB-247, and included within this CUP, will be presented as case reports by physicians, detailing the clinical evolution of every patient as well as the associated immunological data. These preliminary evidences of objective clinical benefits indicate that CIGB-247 could be a potentially promising therapeutic option for the treatment of certain types of cancer.

## Conclusions

The CIGB-247 CUP confirms in a variety of cancer patients, under day-to-day clinical practice conditions in several Cuban medical institutions that CIGB-247 is immunogenic and safe. The results also indicate that CIGB-247 is the only one strategy already tested in humans able to induce a VEGF-specific antibody response. Administration of CIGB-247 induces IgM, IgA and IgG antibodies highly specific to human VEGF. This polyclonal response is able to block the interaction between VEGF with VEGFR2, VEGFR1 and bevacizumab. The immunological properties elicited during the induction phase are conserved at the re-immunization phase by monthly vaccinations. In both phases, VEGF-specific IgG1, IgG2, IgG3 and IgG4 subclasses were found, being IgG4 the predominant subclass after 3 years of chronic vaccination. Immunogenicity and preliminary safety data for immunization with CIGB-247 have been extended to previously untested clinical scenarios. The information unraveled by this study provides rationale for the potential combination of CIGB-247 with other onco-specific treatments of many kinds.

## Methods

Relevant information of all commercially available reagents was provided in Additional file 2.

### Investigational product

The antigen used in this study is a recombinant fusion protein, representative of human VEGF isoform 121 (P64KhVEGF_KDR-_) [15]. The lyophilized antigen was produced in vials of 400 μg by the Development Unit of Center of Genetic Engineering and Biotechnology (CIGB, Havana, Cuba). The antigen was mixed with the adjuvant VSSP, very small sized particles obtained from the *Neisseria meningitides* outer membrane, supplied by the Center of Molecular Immunology (CIM, Havana, Cuba). Both, antigen and VSSP were produced under good manufacturing practices conditions. At the moment of vaccination, one antigen vial was dissolved in pre-calculated amounts of injection water, and the required amount was mixed with the established quantity of VSSP (200 μg), up to a final volume never exceeding 1 mL per injection dose.

### Compassionate use program (CUP)

This CUP study of CIGB-247 was conducted in accordance with the Regulation 63–2012 emitted by the Cuban Regulatory Authority (CECMED) [56]. This investigation was also performed in compliance with the ethical guidelines of the Declaration of Helsinki. All physicians interested in including patients in the CUP were required to contact the main coordinator of the study via e-mail (hernandez.bernal@cigb.edu.cu), attaching the following documents: (1) formal request letter asking the compassionate use of CIGB-247 and indicating that the patient received all available onco-specific therapies without response; (2) summary of the patient’s medical record; (3) approval letter by the institution’s ethics committee. After this, the main coordinator sent every physician the informed consent form and other relevant information about the CIGB-247 (list of references, management of adverse events, safety profile). To participate in the CUP, all patients had to sign the informed consent form, after which the physician submitted the document through e-mail, together with the following information: patient’s full name, age, sex, type of cancer (solid tumor or hematologic malignancy), histopathological diagnosis, and the presence or not of metastases. In the case of child participants, it was mandatory the assent from children (whenever possible) and a written informed consent from both parents and/or legal guardians. Once approved by the main coordinator, the patient was assigned a code, and the procedure of the vaccine preparation, immunization schedule and times of blood sampling were sent to the physician by the main coordinator. Subsequently, the Center of Genetic Engineering and Biotechnology delivered the antigen and the adjuvant to the hospital for treatment of the patient. The physician was obligated to report adverse events, probably or definitively related to the vaccine. The acquisitions of blood samples were not mandatory.

### Patient inclusion criteria and immunization protocol

CUP included subjects of any sex and age, diagnosed with solid tumor or hematologic malignancy in early or advanced stages, with non-measurable or measurable lesions, metastases free patients, or individuals with metastatic disease of any localization. There was no restriction for Eastern Cooperative Oncology Group performance status, for chronic un-compensated diseases, autoimmune or immune suppressing diseases. Patients receiving immune modulator drugs, chemotherapy or biological therapies including active or passive immunotherapy were also recruited. Subjects with allergies to vaccine components, pregnancy or breast feeding, and evident mental incapacity to understand the information, deliver the consent, and act in consequence during the study were excluded.

One hundred fifty three patients were immunized with 400 μg of antigen in combination with 200 μg of the adjuvant VSSP, which represents the highest antigen dose that at that point of the initiation of this program had been found to be safe. All vaccinations were administered subcutaneously as a single site dose. Each patient received eight weekly vaccinations followed by a re-immunization on week 12 (induction phase). Four weeks after the ninth vaccination (week 16), re-immunizations started once every 4 weeks, until death, intolerance, marked disease progression or patient’s withdrawal of consent (re-immunization phase).

### Human blood samples

Blood samples were obtained from 41 of the 153 vaccinated patients. Venous blood samples were collected using a blood collection set with pre-attached holder and taken into a serum separator tube for serum analyses. Serum samples were obtained as previously described [55, 57], and were immediately stored at − 20 °C or − 70 °C until use.

Blood samples were taken before initial vaccination (week 0 or pre-vaccination sample) and during the induction phase or re-immunization phase. For investigations conducted during both phases, blood samples were taken at different time points, depending on patient availability.

### Biotinylation of monoclonal antibody bevacizumab and VEGF binding testing

To develop a competitive ELISA that measures the inhibition of the binding between VEGF and bevacizumab, this recombinant monoclonal antibody was biotinylated. A bevacizumab solution of 3.68 mg/mL was obtained in labelling buffer (0.1 M NaHCO_3_, 0.1 M NaCl pH 8.5) by exchange chromatography. Biotin N-hydroxysuccinimide ester was added to bevacizumab solution at a ratio of 0.1 mg of biotin per mg of antibody. The reaction mixture was incubated with stirring during 4 h at room temperature. Free biotin was removed and antibody was exchanged into phosphate buffer saline (PBS) by gel filtration. VEGF binding curves for bevacizumab and biotinylated bevacizumab yielded similar half maximal effective concentration (EC_50_), indicating that conjugation did not affect the antigen-binding site (Additional file 3).

### ELISAs reagents

Human VEGF isoform 121 (hVEGF _CHO_) and human VEGF-C (hVEGF-C _CHO_) were produced in CHO cells [58]. Plasmid construction and cell line development for hVEGF-C _CHO_ are described in Additional file 4. Horseradish peroxidase (HRP)-conjugated goat anti-human IgG (Fc γ fragment specific) antibody was used at 80 ng/mL for detecting human serum IgG. Biotinylated goat antibodies specific for human VEGFR2 or human VEGFR1 were used at 0.1 μg/mL for detecting the VEGF/VEGFR2 or VEGF/VEGFR1 bindings respectively. Streptavidin-peroxidase conjugate was used 1/30000 or 1/35000 dilution. Human serum, positive (PCS) or negative (NCS) for VEGF-specific IgG antibodies, have been previously used as assay controls [58].

### ELISA for specific anti-human VEGF IgG, IgM, IgA and IgE antibodies

The levels of human IgG, IgM, IgA and IgE antibodies against VEGF were measured as previously described [19, 58]. Briefly, wells were coated with hVEGF_CHO_ during overnight incubation at 4 °C. Following blocking step, the wells were incubated with samples and IgG, IgM, IgA or IgE antibodies were detected with HRP-conjugated goat anti-human IgG antibody, biotinylated goat anti-human IgM antibody, biotinylated anti-human IgA monoclonal antibody or biotinylated anti-human IgE monoclonal antibodies, respectively. For biotinylated conjugates the detection system consisted of streptavidin-conjugated HRP. Plates were developed by using H_2_O_2_ as substrate and TMB as chromogen.

IgG antibody titer was estimated as previously described [58]. The procedure was similar for IgM, IgA and IgE with the difference that the interpolated value on “x” axis was determined by adding five standard deviations to the duplicated mean of the blank optical density.

Titer ratio and “VEGF-specific antibody titer” were calculated as follow:

1 $$ \mathrm{Titer}\ \mathrm{ratio}=\frac{\mathrm{Post}\ \mathrm{vaccination}\ \mathrm{titer}}{\mathrm{Pre}\ \mathrm{vaccination}\ \mathrm{titer}} $$

2 $$ \mathrm{Specific}\ \mathrm{antibody}\ \mathrm{titer}=\mathrm{Post}\ \mathrm{vaccination}\ \mathrm{titer}-\mathrm{Pre}\ \mathrm{vaccination}\ \mathrm{titer} $$

To declare a given sample taken during vaccination to be positive for VEGF-specific IgG, IgM, IgA, or IgE antibodies, the obtained “titer ratio” must be ≥2 (formula A). In the particular case of IgG antibodies, additionally to the criterion depicted above, for a sample to be considered positive, it has also to comply with a value of “specific antibody titer” ≥1/100 (formula B).

The term seroconversion is only used in this paper for IgG antibodies and refers to a patient that has shown two or more samples positive for VEGF-specific antibodies during the re-immunization phase (seroconverted patient) [18].

### IgG subclasses assays

VEGF-specific IgG1, IgG2, IgG3, and IgG4 antibodies were determined as previously described [19]. Briefly, microtiter plates were coated with hVEGF _CHO_ during overnight incubation at 4 °C. Following a blocking step, sera were added and antigen-specific IgG1, IgG2, IgG3, and IgG4 antibodies were detected using biotinylated mouse monoclonal anti-human subclass-specific antibodies.

To declare a given serum sample taken during vaccination as “non-detectable” for VEGF-specific IgG1, IgG2, IgG3, or IgG4 antibodies, the “specific antibody titer” must be < 1/10. Values ≥1/10 make samples to be classified as “detectable”. For each patient, the IgG subclass classified as “detectable” with the highest “specific antibody titer” was declared as “predominant” [19].

### Competitive ELISA evaluating the blockade of the binding between VEGF and its receptors

Competitive ELISA details have been previously described by Sánchez et al. [19, 58]. Briefly, plates were coated with hVEGF _CHO_ during overnight incubation at 4 °C. Following a blocking step, sample was added and incubated for 1 h at 37 °C. Then, 100 μL of 25 ng/mL of VEGFR2-Fc or 125 ng/mL of VEGFR1-Fc were added to the wells (12.5 and 62.5 ng/mL final concentration respectively) and additionally incubated for 45 min at 37 °C. After washes, wells were incubated with biotinylated anti-human VEGFR2 or VEGFR1 antibodies, the latter followed by streptavidin-peroxidase conjugate.

Maximum bindings of VEGFR2 or VEGFR1 were obtained from wells incubated with dilution buffer (instead of sample) and VEGF receptors/Fcγ chimeras (VEGFR2-Fc or VEGFR1-Fc). The inhibition caused by a given sample on VEGF/VEGFR2 or VEGF/VEGFR1 interactions was expressed as percentage, according to the following formula:

3 $$ \%\mathrm{inhibition}=100\%-\left[\left(\frac{\mathrm{absorbance}\ \mathrm{of}\ \mathrm{test}\ \mathrm{sample}}{\mathrm{absorbance}\ \mathrm{of}"\mathrm{Maximum}\ \mathrm{Binding}"}\ \right)\ast 100\right] $$

Inhibition levels were expressed as a % ratio:

4 $$ \mathrm{inhibition}\ \mathrm{levels}=\frac{\mathrm{Post}\ \mathrm{vaccination}\ \mathrm{inhibition}\ \left(\%\right)}{\mathrm{Pre}\ \mathrm{vaccination}\ \mathrm{inhibition}\ \left(\%\right)} $$

A given sample was considered positive for blocking activity when the value resulting from this ratio was ≥2 (formula D). Patients showing at least one serum sample with neutralizing anti-VEGF antibodies during induction phase or re-immunization phase were considered with a positive blocking activity on the VEGF/VEGFR1 or VEGF/VEGFR2 bindings [18].

### Competitive ELISA evaluating the blockade of the interaction between VEGF and bevacizumab

Plates were coated with hVEGF _CHO_ (1 μg/mL in PBS, 100 μL/well, overnight incubation at 4 °C). After three washes with 0.1% Tween 20 in PBS, the plates were blocked with 2.5% goat serum (v/v), 2% skim milk (m/v), 0.05% Tween 20 (v/v) in PBS (250 μL/well, 1 h at 37 °C). After a washing step, test sample or dilution buffer were added (100 μL/well, 1 h at 37 °C). Then, 100 μL/well of biotinylated bevacizumab antibody at 7.6 ng/mL were added to the wells (3.8 ng/mL final concentration and diluted in blocking buffer) and additionally incubated for 1 h at 37 °C. The maximum binding of bevacizumab was obtained from incubated wells with dilution buffer (instead of sample) and biotinylated bevacizumab. After washes, wells were incubated streptavidin-peroxidase conjugate (diluted 1:30000 in 1% bovine serum albumin (BSA)/PBS, 100 μL/well, 45 min at 37 °C). After washes, the subsequent steps of the reaction were developed as described in previous sub-sections.

Each plate included “blank” wells that were developed in parallel and did not receive neither test samples nor biotinylated bevacizumab, only dilution buffer. The other ELISA steps (antigen coating, adding blocking buffer, incubating with biotinylated antibody and streptavidin-peroxidase conjugate, adding substrate and stopping buffer) were performed as those for other wells.

The inhibition caused by a given sample on VEGF/bevacizumab interaction was expressed as percentage, according to formula C. The final concentration of 3.8 ng/mL corresponds to half-maximal effective concentration (EC_50_), and this value was obtained from four independent experiments (Additional file 5).

### ELISA for detecting human IgG antibodies specific to VEGF-C and VEGF-D

Two strategies were used for detecting human IgG antibodies specific to VEGF-C and VEGF-D. For the first one, wells were coated with a monoclonal antibody specific to myc-tagged proteins (10 μg/mL in PBS, 100 μL/well, overnight incubation at 4 °C). Following a washing step (0.12% Tween 20 v/v) and a blocking step (2.5% goat serum v/v, 2% skim milk m/v, 0.05% Tween 20 v/v in PBS, 250 μL/well, 1 h at 37 °C), the wells were incubated with hVEGF-C _CHO_ or hVEGF _CHO_ (5 μg/mL in blocking buffer, 100 μL/well, 1 h at 37 °C). Plates were washed, and test samples were added (diluted in blocking buffer, 100 μL/well, 1 h at 37 °C). Specific IgG antibodies were detected with HRP-conjugated goat anti-human IgG antibody (diluted in 2% skim milk v/v in PBS, 100 μL/well, 1 h at 37 °C). Plates were developed by using H_2_O_2_ as substrate and TMB as chromogen (100 μL/well, 10 min at room temperature). The reaction was stopped by the addition of 2.0 N H_2_SO_4_ (50 μL/well), and the absorbance was measured at 450 nm.

For the second strategy, VEGF-C or VEGF-D (2.5 μg/mL in PBS, 100 μL/well, overnight incubation at 4 °C) were added to Histidine-select nickel coated high sensitivity multiwell plates. After a washing step (0.05% Tween 20 v/v in PBS), test samples were added (diluted in 0.05% skim milk v/v, 0.05% Tween 20 in PBS, 100 μL/well, 2 h at 37 °C), and specific IgG antibodies were detected with HRP-conjugated goat anti-human IgG antibody (diluted in 0.05% skim milk v/v, 0.05% Tween 20 in PBS). The subsequent steps of the reaction were developed as previously described. Recombinant human VEGF receptor 3/Fcγ chimera was used as assay positive control.

### IgG fraction purification

Post-vaccination sera from different patients and positive for VEGF-specific IgG antibodies were pooled, and IgG from serum was purified by protein A. Lipoproteins were removed by adding solid polyvinylpyrrolidine to the serum to a final concentration of 3% (w/v). After 4 h at 4 °C, the sample was centrifuged at 15700 g for 30 min at 4 °C. Supernatant was removed and exchanged into 0.02 M sodium phosphate buffer pH 7 (binding buffer) using a desalting column. After centrifugation, supernatant was mixed to a pre-equilibrated protein A media, and later incubated during 14-16 h at 4 °C with stirring. The gel bed was added to an empty column, and the excess fluid was allowed to drain via gravity. The gel bed was washed with binding buffer, and the IgG fraction was eluted 0.1 M glycine buffer pH 2.7. The IgG fraction was collected into a neutralization buffer (1 M Tris-HCl pH 9). The eluate was immediately exchanged into PBS, concentrated between 10 and 20 mg/mL of IgG, and the final sample was stored at − 70 °C until use (IgG comp). A purified human IgG isolated from pooled normal human serum (IgG neg) was used as assay negative control.

### Statistical analysis

Data, graphs and statistic were analyzed with GraphPad Prism software version 6.0. Two-group comparisons of unpaired data were made using the *t*-test. Statistical significance was considered as *p* < 0.05.

## Supplementary information

**Additional file 1.** VEGF-specific IgG, IgM, IgA and IgE antibodies.

**Additional file 2.** Reagents.

**Additional file 3.** Biotinylation of bevacizumab did not affect the binding to VEGF.

**Additional file 4.** Human VEGF-C from transfected CHO cells.

**Additional file 5.** Half maximal effective concentration (EC50) for biotinylated bevacizumab obtained from four independent experiments.

## Data Availability

All data generated or analyzed during this study are included in this published article in Figs. 1, 2, 3, 4 and 5; Tables 1 and 2; and in its Additional files 1, 2, 3, 4 and 5**.**

## Abbreviations

BSA: Bovine serum albumin
CECMED: Medicines Regulatory Authority, Equipment and Medical Devices of the Republic of Cuba
CUP: Compassionate use program
EC_50_: Half maximal effective concentration
EGF: Epidermal growth factor
ELISA: Enzyme-linked immunosorbent assay
ELISPOT: Enzyme-linked immunospot assay
HER 2-ECD: Extracellular domain of HER 2
HER: Epidermal growth factor receptor
HRP: Horseradish peroxidase
IFN-γ: Interferon-gamma
NCS: Negative control serum
PBMC: Peripheral blood mononuclear cells
PBS: Phosphate buffer saline
PCS: Positive control serum
TGF-α: Transforming growth factor-α
TT: Tetanus toxoid
VEGF: Vascular endothelial growth factor
VEGFR: Vascular endothelial growth factor receptor
