# Supplementary material for: Specific humoral response in cancer patients treated with a VEGF-specific active immunotherapy procedure within a compassionate use program
Source: BMC Immunol. 2020 Mar 14;21:12. doi: 10.1186/s12865-020-0338-4 (PMC7071683; doi:10.1186/s12865-020-0338-4)
Supplement: Supplementary file 2 — Additional file 2. Reagents. [file 12865_2020_338_MOESM2_ESM.docx]

Additional file 2. Reagents.

| **Name** | **Denotation** | **Supplier** | **Catalogue** | **Relevant Information** |
| --- | --- | --- | --- | --- |
| Human VEGF obtained from transfected CHO cells | hVEGF _CHO_ | CIGB, Havana | - | isoform 121, histidine and cmyc-tagged protein |
| Variant of human VEGF-C obtained from transfected CHO cells | hVEGF-C _CHO_ | CIGB, Havana | - | Ala112-Arg227 with the addition of three aminoacids (Ala, Pro, Met), histidine and cmyc-tagged protein |
| Mouse monoclonal antibody specific for c-myc tag | - | CIGB, Sancti Spiritus | CB9E10 | - |
| Human VEGF-C (NS0-derived protein) | hVEGF-C | R&D Systems | 2179-VC | Thr103-Arg227, with a C-terminal 10-His tag |
| Human VEGF-D (Sf 21 baculovirus-derived protein) | hVEGF-D | R&D Systems | 622-VD | Phe93-Ser201, with a C-terminal 6-His tag |
| Human VEGF R2/KDR/Flk‑1 Biotinylated Antibody | - | R&D Systems | BAF357 | - |
| Human VEGF R1/Flt‑1 Biotinylated Antibody | - | R&D Systems | BAF321 | - |
| Skim milk powder | - | AppliChem | A0830 | - |
| Tween 20 | - | AppliChem | A1389 | - |
| HRP-conjugated goat anti-human IgG (Fc γ fragment specific) antibody | - | Jackson Immunoresearch Laboratories | 109-035-098 | - |
| Goat anti-human IgM, biotinylated | - | Mabtech | 3840-6-250 | - |
| anti-human IgA mAb MT20, biotinylated | - | Mabtech | 3860-6-250 | - |
| anti-human IgE mAbs 107/182/101, biotinylated | - | Mabtech | 3810-8-250 | - |
| Biotinylated mouse monoclonal antibodies specific for human IgG1 | - | Abcam | ab9975 | - |
| Biotinylated mouse monoclonal antibodies specific for human IgG2 | - | Abcam | ab99785 | - |
| Biotinylated mouse monoclonal antibodies specific for human IgG3 | - | Abcam | ab99830 | - |
| Biotinylated mouse monoclonal antibodies specific for human IgG4 | - | Abcam | ab99824 | - |
| VEGF Receptor-2 (Flk-1, KDR)/Fc Chimera human | VEGFR2-Fc or VEGFR2 | Sigma | V6758 |  |
| VEGF Receptor-1 (Flt-1)/Fc Chimera human | VEGFR1-Fc or VEGFR1 | Sigma | V1385 | - |
| VEGF Receptor-3 (Flt-4)/Fc Chimera human | VEGFR3-Fc or VEGFR1 | Sigma | V1260 |  |
| Streptavidin−Peroxidase | - | Sigma | S5512 | - |
| (+)-Biotin N-hydroxysuccinimide ester | - | Sigma | H1759 | - |
| IgG from human serum | IgG neg | Sigma | I4506 | Isolated from pooled normal human serum by fractionation and ion-exchange chromatography. |
| Bovine serum albumin | BSA | Sigma | A3294 | - |
| HIS-Select® High Sensitivity (HS) Nickel Coated Plates | - | Sigma | S5688 | - |
| humanized anti-VEGF monoclonal antibody | bevacizumab | Roche | - | - |
| TMB | - | Calbiochem | 613544 | - |
| rProtein A Sepharose® Fast Flow | - | GE Healthcare | 17-1279-02 | - |
| Polyvinylpyrrolidine | - | Merck | 7443 | - |
| CentriPure P100 Columns | - | EMP Biotech | CP-0119 | Protein Desalting Columns |
| Blood collection set with pre-attached holder | - | Becton Dickinson | 367355 | - |
| Phosphate buffer saline pH 7.4 | PBS | Invitrogen | 00300 | - |
