# Supplementary material for: Specific humoral response in cancer patients treated with a VEGF-specific active immunotherapy procedure within a compassionate use program
Source: BMC Immunol. 2020 Mar 14;21:12. doi: 10.1186/s12865-020-0338-4 (PMC7071683; doi:10.1186/s12865-020-0338-4)
Supplement: Supplementary file 3 — Additional file 3. Biotinylation of bevacizumab did not affect the binding to VEGF. [file 12865_2020_338_MOESM3_ESM.docx]

**Additional file 3. Biotinylation of bevacizumab did not affect the binding to VEGF**


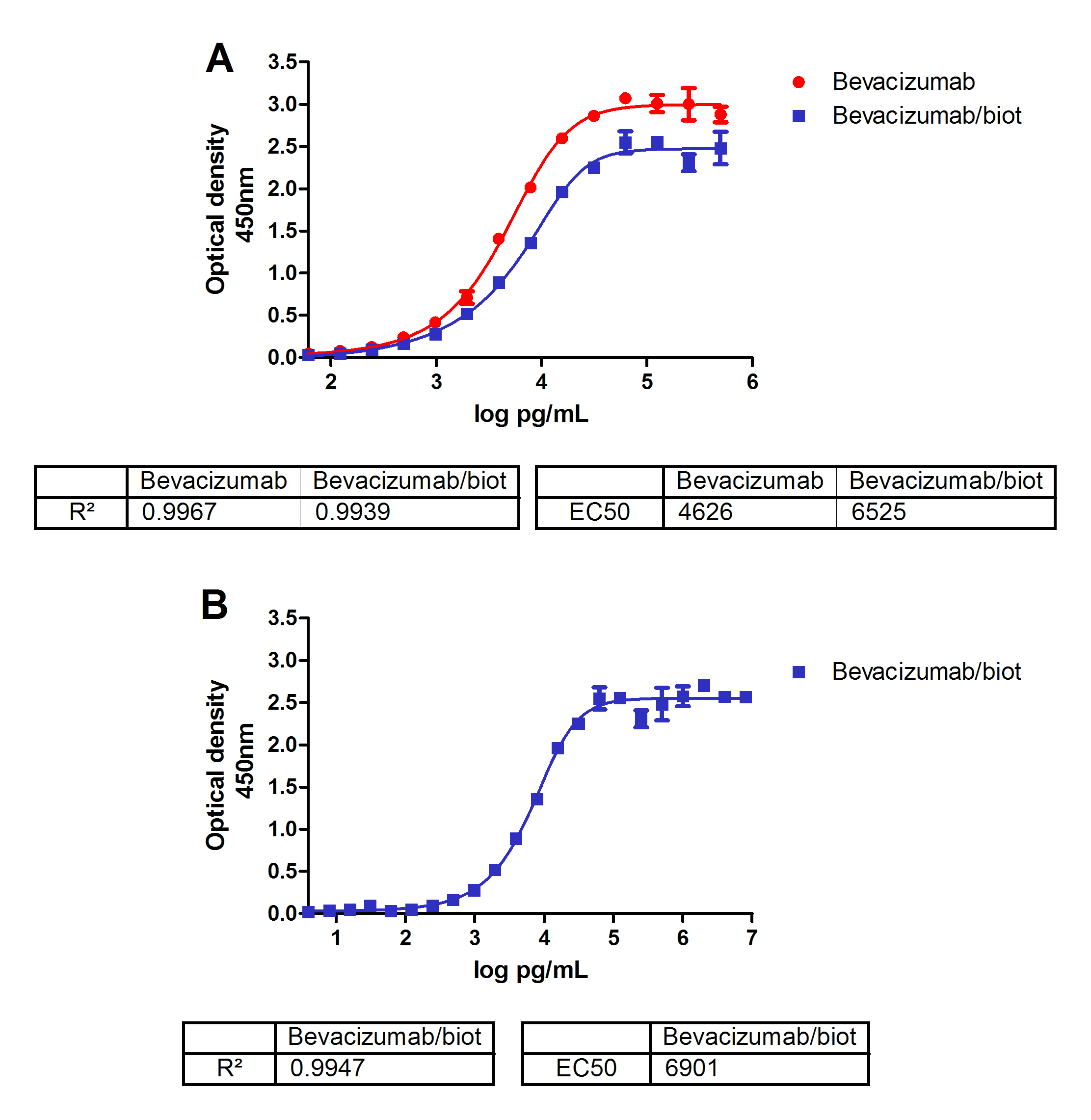


**Binding curves of bevacizumab to human VEGF.** Plates were coated with hVEGF _CHO_ (1µg/mL in PBS, 100µL/well, overnight incubation at 4ºC). After three washes, the plates were blocked for 1h at 37ºC. After a washing step, different concentrations of the monoclonal antibody bevacizumab or biotinylated bevacizumab (bevacizumab/biot) were added (100 µL/well, 1h at 37ºC). (A) The binding of bevacizumab to VEGF was detected with HRP-conjugated goat anti-human IgG antibody. (B) The binding of bevacizumab/biot to VEGF was detected with streptavidin-peroxidase conjugate. The half maximal effective concentration (EC_50_) was calculated using a 5-parameter logistic curve-fitting analysis in GraphPad 6.0 computer software.
