# Supplementary material for: Specific humoral response in cancer patients treated with a VEGF-specific active immunotherapy procedure within a compassionate use program
Source: BMC Immunol. 2020 Mar 14;21:12. doi: 10.1186/s12865-020-0338-4 (PMC7071683; doi:10.1186/s12865-020-0338-4)
Supplement: Supplementary file 4 — Additional file 4. Human VEGF-C from transfected CHO cells. [file 12865_2020_338_MOESM4_ESM.docx]

**Additional file 4. Human VEGF-C from transfected CHO cells**

**Plasmid construction and cell line transfection with a DNA coding for a variant of human VEGF-C (hVEGF-C _CHO_)**

To construct a VEGF-C expressing vector, a gene coding the sequence of human VEGF-C (112-227) was amplified with the 5´ primer (5’-GGGCCATGGCGCATTACAATACCG-3’) and the 3´ primer (5´-GGGGAATTCATTAACGACGAATAATGCTATGCACC-3’) by PCR from a plasmid pPACIB/VEGF-C 112-227. The signal peptide of VEGF was incorporated into the amplified sequence by PCR using the primers 5´-GGGGGATCCCACCATGAACTTTCTGCTGTCTTGGGTG-3´ and 5´-CCATTCGAAACGACGAATAATGCTATGCACC-3´. The obtained DNA was cloned into the BamHI and BstBI sites of pcDNA 3.1/myc-His C vector (Invitrogen, V800-20). The construction of cloned gene was confirmed by DNA sequencing and then transfected into CHO cells (Chinese hamster ovary cells) using Superfect Transfection Reagent (Qiagen, 1006699). Transfected cells were selected for growth in the presence of G418 (Sigma, A8601) and two rounds of single-cell dilution were conducted. After that, clones were adapted in growth medium without antibiotic selector. The finally selected cell clone was chosen by secreted VEGF-C levels per million of cells.

**Harvesting and purification of cell culture supernatant**

Stably transfected cells were cultured in DMEM containing 10% fetal bovine serum and 50µg/mL gentamicin. The cell culture supernatant was collected seven days after reaching 100% of confluence. Ni-NTA resin (Qiagen, 30410) equilibrated with buffer A (20mM Tris, 150mM NaCl pH 7) containing 5mM imidazole was used for the purification of VEGF-C. Cell culture media, previously diluted 1/2 with buffer A was loaded into the column. The column was washed with five column volumes of equilibration buffer followed by three column volumes of buffer A containing 50mM imidazole. Recombinant VEGF-C was eluted with buffer A containing 300mM imidazole. EDTA 5mM was added to the elution fraction and dialyzed against buffer A containing 5mM EDTA. Purified VEGF-C (hVEGF-C _CHO_) was quantified using a commercially available kit (R&D Systems, DVEC00).

**hVEGF-C _CHO_ binds VEGFR2 and VEGFR3 measured by ELISA**


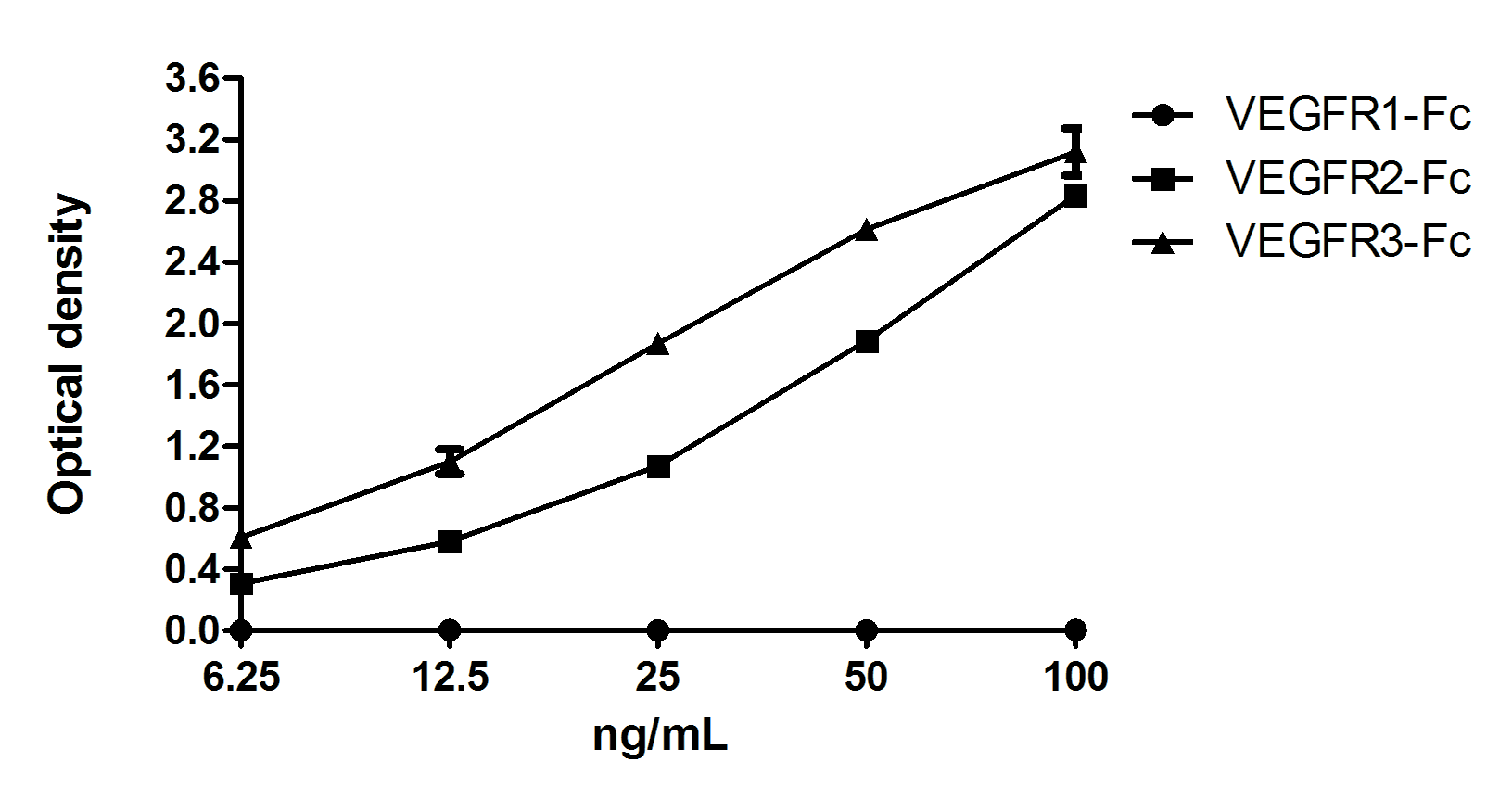


Wells were coated with a monoclonal antibody specific to myc-tagged proteins (10 µg/mL in PBS, 100µL/well, overnight incubation at 4ºC). Following a washing step (0.12% Tween 20 v/v) and a blocking step (2.5% goat serum v/v, 2% skim milk m/v, 0.05% Tween 20 v/v in PBS, 250µL/well, 1h at 37ºC), the wells were incubated with hVEGF-C _CHO_ (5 µg/mL in blocking buffer, 100µL/well, 1h at 37ºC). Plates were washed, and VEGF receptors/Fcγ chimeras (VEGFR2-Fc or VEGFR3-Fc) were added (diluted in blocking buffer, 100µL/well, 1h at 37ºC). Specific IgG antibodies were detected with HRP-conjugated goat anti-human IgG antibody (diluted in 2% skim milk v/v in PBS, 100µL/well, 1h at 37ºC). Plates were developed by using H_2_O_2_ as substrate and TMB as chromogen (100 µL/well, 10 minutes at room temperature). The reaction was stopped by the addition of 2.0 N H_2_SO_4_ (50 μL/well), and the absorbance was measured at 450nm. VEGFR1-Fc was used as assay negative control.
