# Supplementary material for: Specific humoral response in cancer patients treated with a VEGF-specific active immunotherapy procedure within a compassionate use program
Source: BMC Immunol. 2020 Mar 14;21:12. doi: 10.1186/s12865-020-0338-4 (PMC7071683; doi:10.1186/s12865-020-0338-4)
Supplement: Supplementary file 5 — Additional file 5. Half maximal effective concentration (EC50) for biotinylated bevacizumab obtained from four independent experiments. [file 12865_2020_338_MOESM5_ESM.docx]

**Additional file 5. Half maximal effective concentration (EC50) for biotinylated bevacizumab obtained from four independent experiments.**


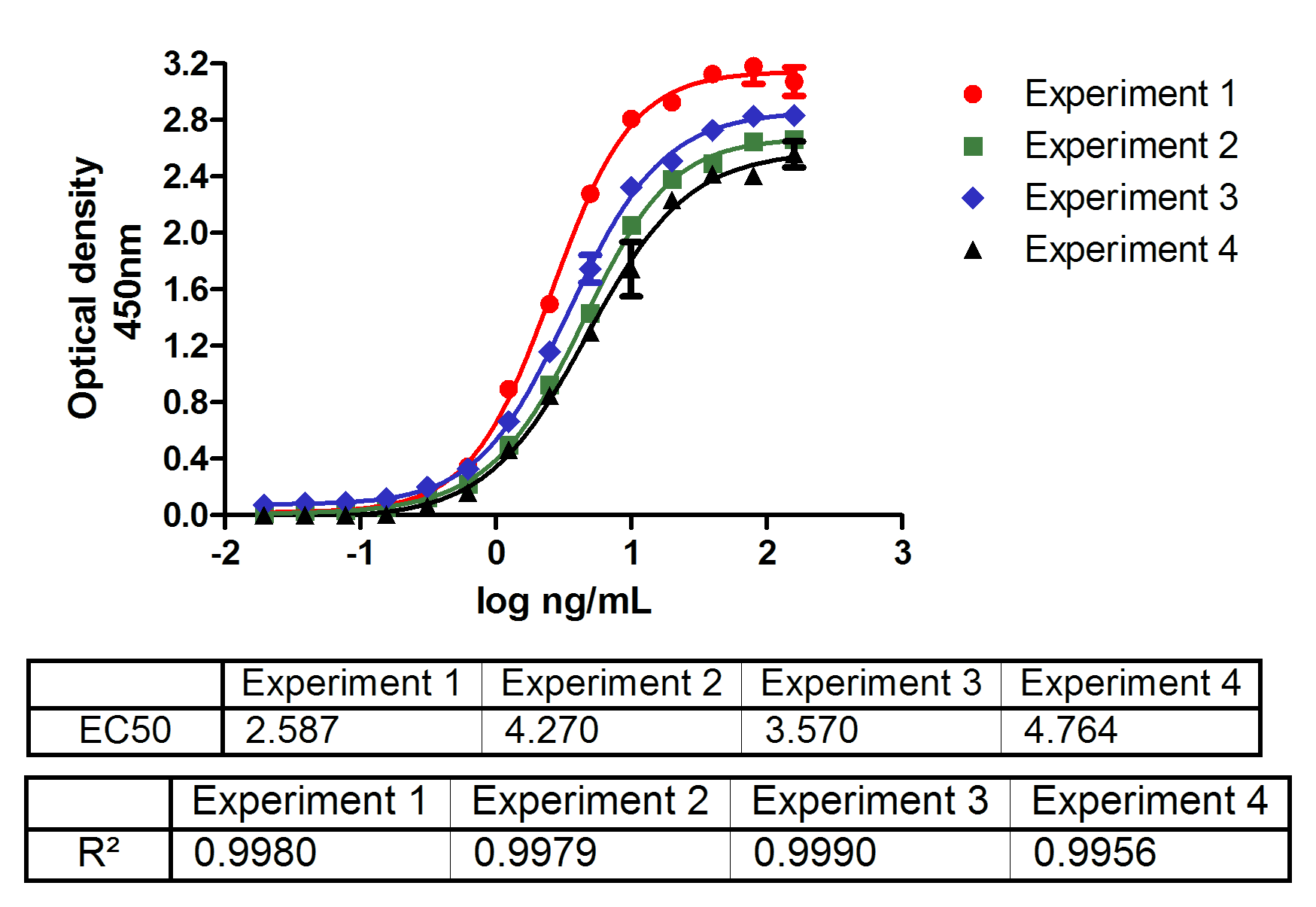


**Binding curves of biotinylated bevacizumab to human VEGF.** Plates were coated with hVEGF _CHO_ (1µg/mL in PBS, 100µL/well, overnight incubation at 4ºC). After three washes, the plates were blocked for 1h at 37ºC. After a washing step, different concentrations of the biotinylated-monoclonal antibody Bevacizumab were added (100 µL/well, 1h at 37ºC). The binding of bevacizumab/biot to VEGF was detected with streptavidin-peroxidase conjugate. The half maximal effective concentration (EC_50_) was calculated using a 5-parameter logistic curve-fitting analysis in GraphPad 6.0 computer software. The EC_50_ mean value obtained from four independent experiments was equals to 3.8 ng/mL.
